# Three-Dimensional Preoperative Planning Software for Hip Resurfacing Arthroplasty

**DOI:** 10.3390/bioengineering10080939

**Published:** 2023-08-07

**Authors:** M. Abdulhadi Alagha, Kartik Logishetty, Ciaran O’Hanlon, Alexander D. Liddle, Justin Cobb

**Affiliations:** MSk Lab, Department of Surgery and Cancer, Faculty of Medicine, Imperial College London, London W12 0BZ, UK

**Keywords:** hip resurfacing, preoperative planning, 3D planner, arthroplasty

## Abstract

Three-dimensional planning of hip arthroplasty is associated with better visualisation of anatomical landmarks and enhanced mapping for preoperative implant sizing, which can lead to a decrease in surgical time and complications. Despite the advantages of hip resurfacing arthroplasty (HRA), it is considered a technically challenging procedure and associated with inaccurate implant placement. This study aimed to examine the validity, reliability, and usability of preoperative 3D Hip Planner software for HRA. Fifty random cases of various hip osteoarthritis severity were planned twice by two junior trainees using the 3D Hip Planner within a one-month interval. Outcome measures included femoral/cup implant size, stem-shaft angle, and cup inclination angle, and were assessed by comparing outcomes from 2D and 3D planning. An adapted unified theory of acceptance and use of technology (UTAUT) survey was used for software usability. Bland–Altman plots between 3D and 2D planning for stem-shaft and inclination angles showed mean differences of 0.7 and −0.6, respectively (r = 0.93, *p* < 0.001). Stem-shaft and inclination angles showed inter-rater reliability biases of around −2° and 3°, respectively. Chi-square and Pearson’s correlation for femoral implant size showed a significant association between the two assessors (r = 0.91, *p* < 0.001). The 3D test–retest coefficient of repeatability for stem-shaft and inclination angles were around ±2° and ±3°, respectively, with a strong significant association for femoral implant size (r = 0.98, *p* < 0.001). Survey analyses showed that 70–90% agreed that 3D planning improved expectancy in four domains. 3D hip planner appears to be valid and reliable in preoperative HRA and shows significant potential in optimising the quality and accuracy of surgical planning.

## 1. Introduction

Hip resurfacing arthroplasty (HRA) is a more bone-conserving alternative to total hip arthroplasty (THA). It is primarily recommended for younger, active patients with end-stage hip arthrosis [1], and delivers significant biomechanical and functional gains over THA [2,3,4] and a better safety profile [5]. However, HRA comes with a unique complication profile, including femoral neck fracture [6], while metal-on-metal devices are at risk of metal debris and adverse local tissue reactions [7]. Both have been strongly associated with errors of bone preparation and implant position [8,9]. Compared to THA, HRA is considered more technically challenging, with a narrower margin of error.

Conventional pre-operative methods of planning hip arthroplasties use a standing plain radiograph templating of the hip joint to determine cup and femoral stem sizes, inclination angle, neck-shaft angle, and the depth of the cup [10,11,12]. The two-dimensional nature of templating to represent 3D bony structures was shown to be of limited accuracy and reliability in comparison with three-dimensional (3D) planning [13]. Emerging 3D platforms have been developed to plan THA and involve the use of computed tomography (CT) images to construct the plan [14,15]. 

Three-dimensional planning permits clearer visualisation of patients’ unique anatomical landmarks and provides a better map for preoperative implant sizing, which may reduce intraoperative guesswork and translate to a decrease in surgical time and complications [12,16,17]. This is particularly useful to less experienced surgeons as an essential tool to facilitate preoperative mental rehearsal, execution of the procedure and to prevent unanticipated problems [18,19]. Most planning software also measures values such as femoral offset, cup orientation, femoral neck anteversion, and sagittal pelvic tilt [20,21,22,23], allowing surgeons to anticipate potential intraoperative complications (e.g., periprosthetic fractures, implant instability, and leg length discrepancy) [15,23]. It also enables surgeons to reduce surgical device inventory and rationalise their choice of intraoperative equipment [16], yielding more cost-effective surgeries [21]. 

The evolution of 3D planning has acted as a path to introducing innovative assisted technologies in hip surgery, particularly patient-specific instrumentation [24], but also navigation techniques and robotic-assisted surgeries [17]. Implant modifications, such as the introduction of modern ceramics and crosslinked polyethylene, have significantly improved arthroplasty bearing wear with encouraging preliminary results [25]. Similarly, implants which are anatomically matched but have rotational control have been developed, such as ADM; Stryker, Mahwah, NJ; and the H1 cup; Embody Orthopaedic Ltd., London, UK; and 3D planning is part of the workflow for arthroplasty (e.g., Mako SmartRobotics, Stryker; ROSA, Zimmer Biomet; and VELYS, DePuy), all of which is of relevance to the modern surgeon.

Overall, there is sufficient evidence to suggest that 3D planning enhances the precision of arthroplasties [22]. This study sought to examine the validity and reliability of a preoperative 3D Hip Planner software for hip resurfacing arthroplasty and address some of the potential challenges and needs associated with its implementation through survey analyses using an adapted unified theory of acceptance and use of technology (UTAUT) questionnaire.

## 2. Materials and Methods

**Figure 1:**
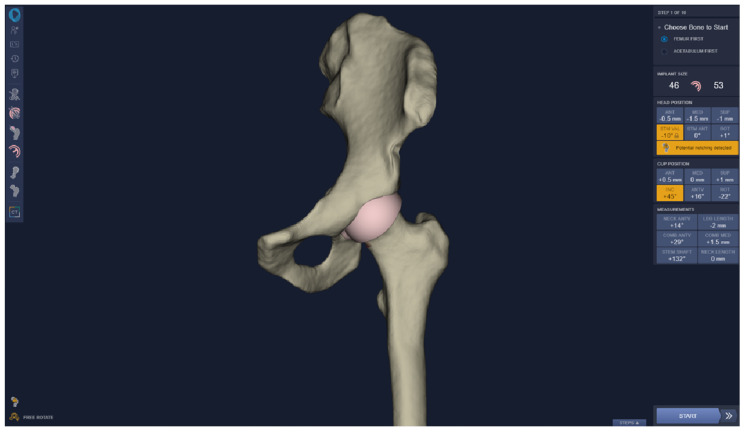
3D Hip Planner software.

Two trainees with limited prior experience in hip arthroplasty planning underwent a three-hour structured training on hip resurfacing procedural elements planning using the 3D Hip Planner (Supplementary Materials S1) and TraumaCad 2D templating software (Version 2.5), based on the recommendations of Solver, Wetter, and Malchau [11] (Supplementary Materials S2). Fifty random surgical cases of varying osteoarthritis severity, statistically powered by the Bland–Altman method [26], were planned twice using the 3D Hip Planner (Figure 1) to examine inter- and intra-rater reliabilities with a minimum of a one-month interval to avoid carry-over effects from the test–retest study design. Concurrent validity against 2D templating was assessed by comparing the outcome measures from the 3D planner and from the 2D system. Software usability was evaluated by engaging clinicians to share their feedback on the usage of the software across five domains using a survey.

### 2.1. Comparison of Techniques (3D vs. 2D)

**Figure 2:**
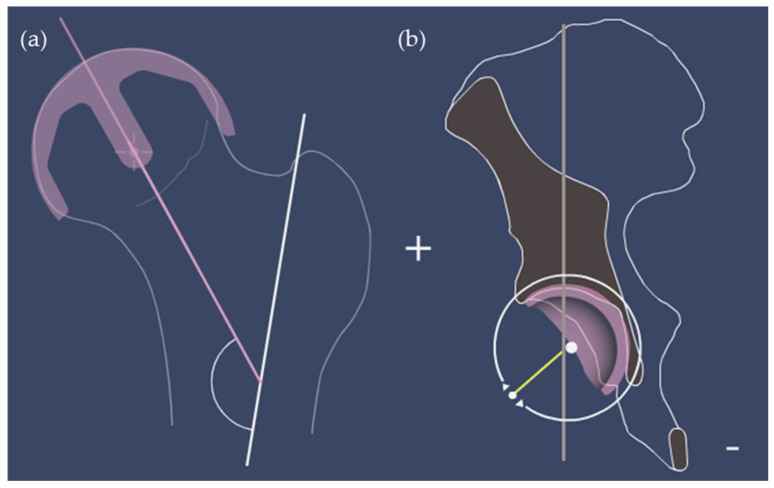
Visual representation of (**a**) stem-shaft angle and (**b**) cup inclination using Embody 3D hip planner software.

Outcome measures were selected by considering the common elements between the two software planning tools, and included femoral/acetabular implant size, stem-shaft angle (Figure 2a), and cup inclination angle (Figure 2b). A visual representation of the 3D hip planner software showing a femur and hemi-pelvis is shown in Figure 1. Statistical analyses were conducted using SPSS version 22. A bivariate two-tailed Pearson’s correlation coefficient (PCC) was computed to measure the strength of the relationship between variables. Bland–Altman plots were used to assess the agreement between 3D and 2D planning as well as estimate the interrater reliability (surgeon 1 vs. surgeon 2) of 3D Hip planner for stem-shaft and inclination angles. Intra-rater (test–retest) reliability for the stem-shaft and inclination angles was examined with the coefficient of repeatability (CR). A chi-squared test was used to measure the agreement of the categorical implant size variable. 

### 2.2. Usability

**Figure 3:**
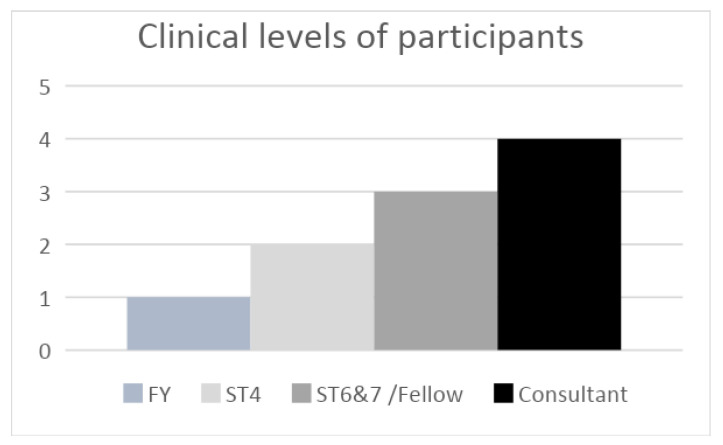
The clinical backgrounds of doctors participating in the adapted UTAUT questionnaire.

Ten medical doctors of various clinical backgrounds (Figure 3) were recruited through convenience sampling to examine the usability of the software. A questionnaire was developed using elements from the unified theory of acceptance and use of technology (UTAUT) validated model [27] and encompassed five domains: performance expectancy, effort expectancy, social influence, facilitating conditions, and attitudes toward 3D planning.

## 3. Results

### 3.1. Agreement with Current Practice

**Figure 4:**
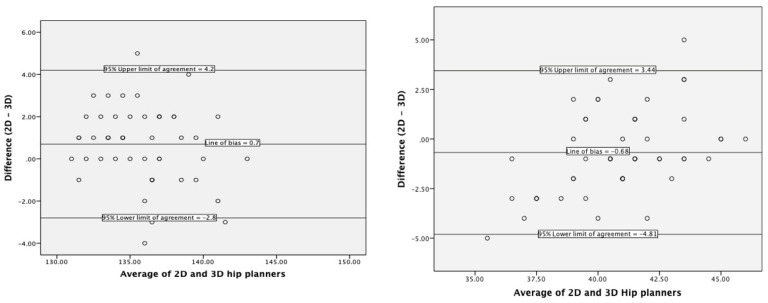
Limits of agreement for stem-shaft (**left**) and inclination (**right**) angles between 3D Hip Planner and 2D templating.

Bland–Altman plots for stem-shaft and inclination angles (Figure 4) showed mean differences of 0.7 (95% upper and lower limits of agreement were −2.8 and 4.2) and −0.6 (−4.8 and 3.4), respectively. There was a high positive correlation between the 3D hip planner and 2D templating for stem-shaft angle (Pearson’s r = 0.83, *p* < 0.001) and inclination angle (Pearson’s r = 0.71, *p* < 0.001).

With regard to femoral implant size, there was a significant association between the 3D hip planner and 2D templating (Χ2(25) = 153.7, *p* < 0.001; Pearson’s r = 0.93, *p* < 0.001).

### 3.2. Interrater Reliability

**Figure 5:**
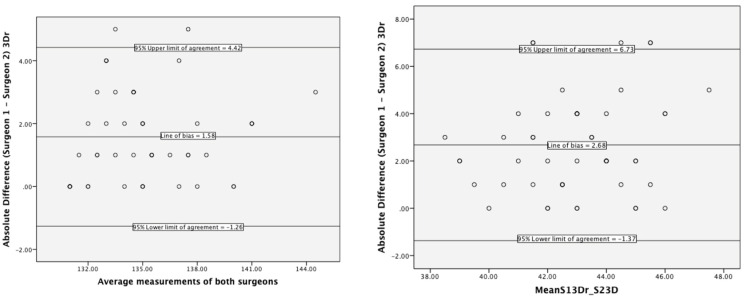
Limits of agreement for stem-shaft (**left**) and inclination (**right**) angles between the two assessors.

Bland–Altman plots for stem-shaft and inclination angles (Figure 5) showed biases of around −2° (95% upper and lower limits of agreement were −1° and 4°) and 3° (1.37 and 6.73), respectively. The chi-square and Pearson’s correlation for femoral implant size showed a significant association between the two assessors (Χ2(30) = 129.3, *p* < 0.001; Pearson’s r = 0.91, *p* < 0.001).

### 3.3. D Test–Retest Reliability

**Figure 6:**
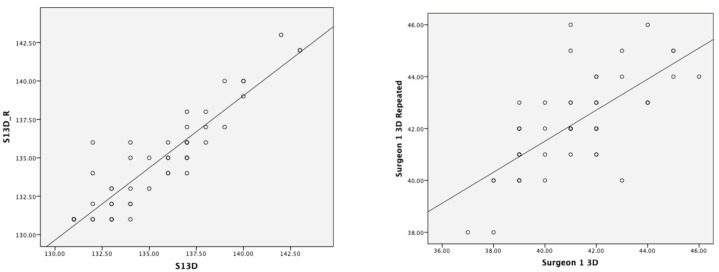
Test–retest Pearson’s correlation for stem-shaft (**left**) and inclination (**right**) angles.

Figure 6 shows stem-shaft and inclination angles measured by the 3D hip planner in the first test compared to the second test (stem-shaft angle Pearson’s r = 0.91, *p* < 0.001; inclination angle Pearson’s r = 0.69, *p* < 0.001). The CR for the stem-shaft angle was around ±2°, with a mean bias of 1.2. The CR for inclination angle was ±3°, with a mean bias of 1°. There was a strong significant association during retest measurements for femoral implant size (Χ2(25) = 217, *p* < 0.001; Pearson’s r = 0.98, *p* < 0.001).

### 3.4. Usability

**Figure 7:**
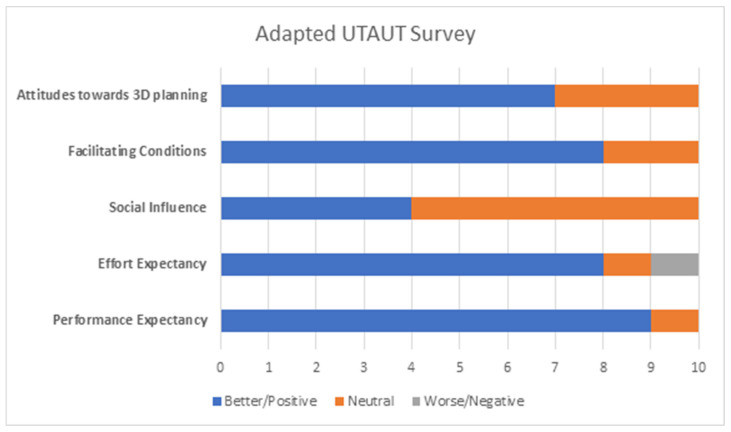
Findings from the adapted UTAUT survey, showing participants’ experience in five domains: performance expectancy, effort expectancy, social influence, facilitating conditions, and attitudes toward 3D planning.

**Figure 8:**
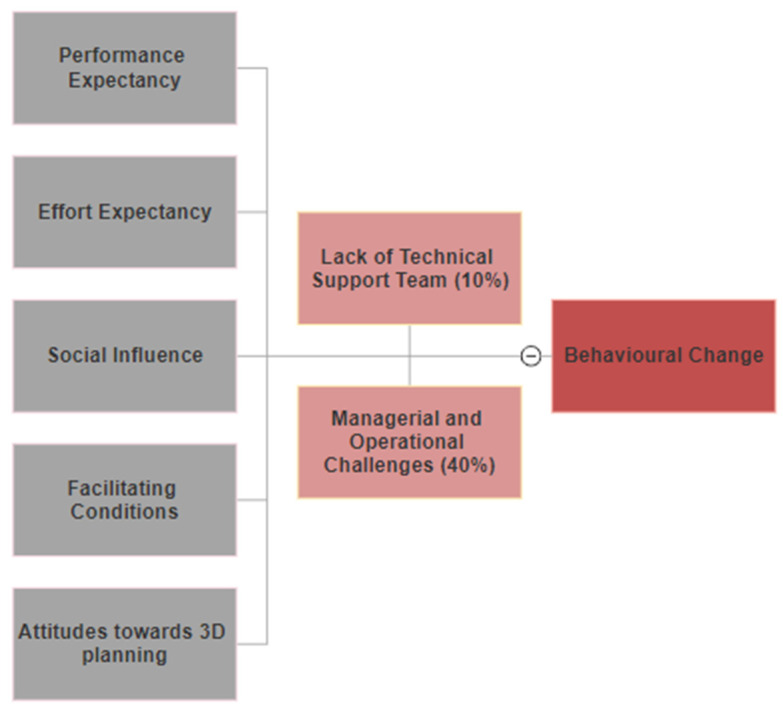
The adapted UTAUT model for 3D hip planning.

Adapted UTAUT survey analyses (Figure 7) of 10 surgeons showed that 90% agreed that 3D planning improved the performance and quality of surgical planning (performance expectancy), with eight participants believing it is easy to use (effort expectancy) with clear identification of anatomical landmarks (facilitating conditions). Similarly, seven participants believed that 3D planning is accurate and interesting and would therefore recommend it to other surgeons. However, only four participants thought that their colleagues would be supportive to use three-dimensional hip planning, partly due to challenges associated with operational implementation and the availability of technical support teams (Figure 8). 

## 4. Discussion

This study sought to examine the validity, reliability, and usability of the 3D Hip Planner in planning hip resurfacing arthroplasty. The findings suggest that 3D Hip Planner software is valid and reliable for hip resurfacing arthroplasty planning and may, as per the UTAUT model, enhance surgical planning.

### 4.1. Agreement with Current Practice 

Compared with 2D templating, the 3D Hip Planner showed good agreement. The findings highlight that stem-shaft and inclination angles may differ by less than one degree if planned in either software. The symmetric spread of data points across the Bland–Altman plots suggests no systematic difference across the range of stem-shaft and inclination angles. Likewise, the strong association of the choice of implant size between the two software (Pearson’s r = 0.93, *p* < 0.001) indicates that clinicians are unlikely to choose a different implant size when planning HRA in three-dimensions compared to 2D.

### 4.2. Interrater Reliability of 3D Hip Planner

The significant association of implant size (Pearson’s r = 0.91, *p* < 0.001) for interrater reliability is higher than previous 2D templating studies [k = 0.25–0.32 [28]; and k = 0.22–0.43 [29]] and indicates that 3D planning is an appropriate sizing tool for HRA. Biases for interrater stem-shaft and inclination angles were −2° and +3°, respectively, with an equal spread of data points and relatively narrow limits of agreements. This translates into differences among assessors in planning these angles to be different by about 1.5° for stem-shaft angle and less than 3° for inclination angle. 

It is worth noting that 2D planning was shown to be accurate and reliable in preoperative THA templating [30] and HRA planning [31]. The authors compared 2D templating to computed tomography, which was assumed to be a true representation of implant orientation. Previous studies in THA demonstrated the excellent reliability of CT-based 3D planning for implant size and alignment [32,33]. This study shows comparable findings with high levels of reliability for the 3D planning of HRA. 

### 4.3. Test–Retest Reliability of 3D Hip Planner

There was a strong correlation for implant size during retest measurements (Pearson’s r = 0.98, *p* < 0.001). There appeared to be modest variability in the repeated 3D stem-shaft and inclination angles measurements as well as between assessors. Two-dimensional templating studies showed high variability among surgeons [k = 0.16–0.73 [28] and k = 0.39–0.61 [29]]. The coefficient of repeatability in our study was less than 2° for stem-shaft angle and less than 3° degrees for inclination angle during intrarater tests. For any observed difference to be considered real, they should be at least as large as CR. Stem-shaft and inclination angles measurements would need to differ by more than ~2° and ~3°, respectively, to reflect a significant difference. This provides the potential implications for the use of 3D planning in HRA. It is unlikely that these CRs reflect carry-over inherent biases or carry-over effects from the study design, since assessors were blinded to their previous outcome measures and a one-month time interval was ensured between the two tests. Statistically, there is no single statistical approach to help researchers decide the magnitude of acceptable CR, it is rather best judged according to its clinical context and relevance [34,35]. Therefore, the minimum clinically important difference (MCID) needs to be measured in clinical settings [36]. Limited evidence exists regarding the limits of agreement for 3D hip resurfacing planning. Mast and colleagues studied the reliability agreement between repeated measures, as given by minimal detectable change, of radiographic parameters on standardised digital anteroposterior and cross-table lateral radiographs, and reported values for neck-shaft angle as 12.2° (interrater reliability) and 4.8°–15.9° (intrarater reliability). Taken together, our findings highlight that 3D planning provides a more robust tool for hip resurfacing arthroplasty.

### 4.4. Usability of 3D Hip Planner

Our adapted UTAUT analyses demonstrated physicians’ views towards 3D hip planning primarily in improving the performance and quality of surgical planning. Two potential barriers to the implementation of 3D Hip Planner were highlighted: lack of technical support teams and managerial/operational challenges. Although participants found positive effort expectancy and facilitating conditions in using three-dimensional planning, a necessary step in preoperative planning is the extraction of anatomical landmarks and reference points. In addition, 3D planning entails an additional annual cost to hospitals, and Huppertz et al. reported an estimated direct cost of 3D preoperative THA planning of EUR 53–116 per patient [37], but the total cost of THA may be reduced by up to 25.7% through automatic selection of hip implants [17,38]. This requires the availability of managerial support, technical engineers, and medical personnel to enhance the learning curve and compensate for barriers to adoption [39]. 

There are a few limitations to report. The fifty randomly selected cases reflected patients with various degrees of hip osteoarthritis severity and did not include cases related to other morphologies such as rheumatoid arthritis or slipped capital femoral epiphysis. Future studies are required to address the suitability of the 3D Hip Planner for different patient cohorts. The two junior assessors had no prior experience in 2D templating and 3D planning, which may yield different findings if senior surgeons were to plan the fifty cases. However, previous studies found similar efficacy of preoperative templating by junior trainees as their senior counterparts [40]. Likewise, 3D planning is believed to be an essential tool to improve mental rehearsal and surgical execution by less experienced surgeons [18], thus it makes it a legitimate cause for carrying out this key validation step by junior trainees. Finally, convenience sampling was used to understand physicians’ attitudes towards using 3D HRA planning. This sampling strategy may be biased towards surgeons who favour the use of surgical technologies in their practice. Large-scale studies should seek to understand and address a wide range of users’ expectations and investigate the benefits of 3D planning in terms of intraoperative performance.

## 5. Conclusions

3D Hip Planner appears to be valid and reliable in preoperative hip resurfacing arthroplasty and shows great potential in optimising the quality and accuracy of surgical planning. It may reduce intraoperative guesswork and complications while arguably enhancing mental rehearsal and learning curves for junior surgeons. The accuracy of templating methods needs to be evaluated by comparing the projected size and position of components with the actual placements during the operative procedure. Prospective clinical studies should address these advantages alongside other measures such as cost-effectiveness, radiation dose associated with the prerequisite low-dose CT scans required for constructing 3D plans, and postoperative outcomes. Likewise, a better understanding of the drivers and barriers to implementation is needed to permit a widespread adoption of this technology in clinical practice.

## Data Availability

The data presented in this study are available on request from the corresponding author. The data are not publicly available due to privacy and ethical restrictions.

## Supplementary Materials

The following supporting information can be downloaded at: https://www.mdpi.com/article/10.3390/bioengineering10080939/s1, Supplementary Materials S1: Steps to plan hip resurfacing cases using 3D Hip Planner; Supplementary Materials S2: Steps to template surgical cases using 2D TraumaCad.
