# Supplementary material for: Three-Dimensional Preoperative Planning Software for Hip Resurfacing Arthroplasty
Source: Bioengineering (Basel). 2023 Aug 7;10(8):939. doi: 10.3390/bioengineering10080939 (PMC10451941; doi:10.3390/bioengineering10080939)
Supplement: Supplementary file 1 [file bioengineering-10-00939-s001.zip › bioengineering-2449185-supplementary.pdf]

Supplementary Materials S1: Steps to plan hip resurfacing cases using 3D Hip Planner

Low dose computed tomography (CT) scans are a prerequisite to construct the 3D plans. 3D planning procedural elements included the following steps.

Step 1: Visualisation in 2D and 3D

Visualise the radiographs in 2D and 3D and identify the presence of osteophytes, dysplastic hips, CAM/Pincer morphology, and avascular necrosis of head of femur. Identify the centre of the head of femur (HoF) while taking into consideration the patient's hip morphology.

Step 2: Femoral Stem Planning

After you identify the centre of the head, select the appropriate size and orientation of the femoral stem implant by placing the base of the implant at the femoral head neck junction. Femoral stem sizes can range from 44 to 54. An adequate size is one with appropriate femoral head coverage while avoiding notching of the femoral neck. Although avoiding notching is important, please note that this might be influenced by the presence of osteophytes. The size should be chosen by appropriately fitting the stem to the femoral head as described above. With regards to stem-shaft angle, slight valgus orientation is recommended. The stem-shaft angle, the angle between the vertical orientation line extending from the femoral stem and the vertical line of the femoral shaft, should be between than 130° and 150°. Adjust the anteversion degree as you see fit and to avoid notching.

Step 3: Acetabular Cup Planning

Medialise (choose the depth of the cup) the acetabular cup taking into account (a) adequate leg-length difference; (b) cup fit and adequate contact with the acetabulum; and (c) avoiding breaching the pelvis. Acetabular cup size is fixed to seven sizes larger than the femoral component. If you adjust the cup size, please ensure that the adjusted femoral stem implant is appropriate. Choose cup inclination angle between 30° and 50° and cup anteversion between 10° and 30°.

Step 4: Review your plan

Visualise the complete preoperative planning of the femoral and acetabular components, where you can modify any sizes and orientations. You are able here to change any of the variables selected above. Once the planning is final, save the plan and take the following measurements:

- Femoral implant size;
- Stem-shaft angle;
- Cup size (7 degrees larger than femoral size);
- Inclination angle.

Supplementary Materials S2: Steps to template surgical cases using 2D TraumaCad

Templating of Hip Resurfacing Arthroplasty in TraumaCad:
These recommendations are drawn from (Solver, Wetter, and Malchau, 2009)

A single anterior–posterior radiograph of the pelvis is used to template hip resurfacing using TraumaCad. Planning starts by noting any pelvic tilt or radiographic abnormalities, such as inadequate radiographs and magnification, that can limit planning.

Step 1: Establish leg-length discrepancy
This is done by drawing a horizontal line, also called a reference line, across the inferior border of pelvic tear drops. Differences in leg length is determined by comparing the vertical lines from each lesser trochanter to the reference line. It is noteworthy that large leg length discrepancies may not be correctable with HRA and might require total hip arthroplasty instead. Because correcting leg length is not an objective of our study, cases were not excluded based on this criterion.

Step 2: Templating femoral component
This step involves two stages: selecting the size and orientation of the implant. Femoral size is first selected by placing the base of the cap at the femoral head neck junction. An adequate size is one with appropriate femoral head coverage while avoiding notching of the femoral neck.
With regards to positioning, neutral or slight valgus orientation should be chosen to avoid femoral neck fractures associated with varus positioning. The stem-shaft angle, the angle between the vertical orientation line extending from the femoral stem and the vertical line of the femoral shaft, should be more than 130 degrees (valgus).

Step 3: Templating acetabular component
The acetabular cup size should have appropriate coverage of the medial wall of the pelvis where it sits with lack of impingement. For an adequate fit with the femoral component, the cup should always be seven sizes larger than the femoral component. The inclination angle of the cup is the angle between the vertical orientation line that extends from the cup and the reference line. To minimise wear rates, the inclination angle should be selected to be between 30 and 50 degrees.
The cup should be oriented with adequate anteversion and vertically between 10 and 30 degrees. This is important to avoid acetabular impingement, wear of bearings, and to ensure hip stability postoperatively and avoidance of impingement.

Step 4: A complete planning
Visualise the complete preoperative planning of the femoral and acetabular components, where you can modify any sizes and orientations. Once the planning is final, save the image and take the following measurements:

- Femoral implant size;
- Stem-shaft angle;
- Cup size (7 degrees larger than femoral size);
- Inclination angle.
